# Lactate dehydrogenase and caspase activity in nasopharyngeal secretions are predictors of bronchiolitis severity

**DOI:** 10.1111/irv.12276

**Published:** 2014-08-12

**Authors:** Reena Mehta, Margaret Scheffler, Lorena Tapia, Letisha Aideyan, Kirtida D Patel, Alan M Jewell, Vasanthi Avadhanula, Minghua Mei, Roberto P Garofalo, Pedro A Piedra

**Affiliations:** a Department of Pediatrics, University of Texas Medical BranchGalveston, TX, USA; b Department of Pediatrics, Baylor College of MedicineHouston, TX, USA; c Department of Molecular Virology and MicrobiologyHouston, TX, USA

**Keywords:** Bronchiolitis, caspase, disease severity, lactate dehydrogenase

## Abstract

**Background:**

Bronchiolitis is the leading cause of hospitalization in infants. Biomarkers of disease severity might help in clinical management.

**Objective:**

To determine the clinical predictiveness of NW-LDH, NW-caspase 3/7, and NW-LDH/NW-caspase 3/7 ratio in bronchiolitis.

**Methods:**

Previously healthy children less than 24 months of age with bronchiolitis were recruited from the Texas Children's emergency room and intensive care unit from October 2010 to April 2011. Demographic, clinical information, and NW samples were obtained at enrollment. NW samples were analyzed for respiratory viruses, caspase 3/7, and LDH.

**Results:**

A viral pathogen was detected in 91·6% of 131 children, with the most common being respiratory syncytial virus and human rhinovirus. A single infection was found in 61·8% of subjects and co-infection in 29·8%. Children admitted to ICU had significantly higher NW-LDH than children sent home from the ER or admitted to the general floor (*P* = 0·02). Children infected with RSV had the highest NW-LDH concentration (*P* = 0·03) compared with other viral infections. NW-LDH and NW-caspase were significantly correlated (*r* = 0·77, *P* < 0·0001). The univariate models showed NW-LDH and NW-LDH/NW- caspase 3/7 ratio were directly associated with hospitalization. Mutivariate regression analyses suggested a complex interaction between the biomarkers, demographics, and disposition.

**Conclusions:**

NW-LDH, NW-caspase 3/7 and NW-LDH/NW-caspase 3/7 ratio and their interactions with demographic factors are predictive of bronchiolitis severity and can help distinguish children requiring ICU-level care from those admitted to the general floor, or discharged home from the emergency center.

*Please cite this paper as:* Mehta *et al*. (2014) Lactate dehydrogenase and caspase activity in nasopharyngeal secretions are predictors of bronchiolitis severity. Influenza and Other Respiratory Viruses 8(6), 617–625.

## Background

Bronchiolitis is the commonest lower respiratory tract illness in young children and the leading cause of hospitalization in this age group in the United States, resulting in significant morbidity and mortality in children less than 2 years.^1^,^2^ Bronchiolitis is primarily a viral illness, with respiratory syncytial virus (RSV) followed by rhinovirus (RV) as the most common viral etiologies.^3^–^7^ The clinical spectrum of disease varies from mild illness not requiring hospitalization to severe respiratory failure necessitating ventilatory support in the intensive care unit. In the first year of life, approximately 15–20% of children with RSV will seek medical attention.^8^ The majority of these children (95%) are treated as outpatients, in primary care physician offices or the emergency department.^8^ Young age and co-morbidities such as prematurity, congenital heart disease, neuromuscular disease, and immunodeficiency are important risk factors for hospitalization,^9^,^10^ but determining the severity of disease can be still difficult in young infants and methods often differ among institutions.^11^ Bronchiolitis is a clinical diagnosis and currently no standardized methods exist to aid the physician in determining the disposition of a patient. Clinicians commonly rely on parental history, clinical findings and the presence or absence of hypoxemia. Molecular diagnostics have improved our understanding of the viral etiology of bronchiolitis and the common occurrence of viral co-infections,^12^ but there remains a clinical need for predictive biomarkers that can aid clinicians in the management and disposition of their patients with bronchiolitis.

Recently, we observed that nasal wash lactate dehydrogenase (NW-LDH) was a good predictor of bronchiolitis severity.^13^ NW-LDH was inversely correlated with disease severity in infants and young children presenting to the emergency department with bronchiolitis.^13^ It also significantly correlated with NW-caspase 3/7 activity, which is a marker of apoptosis, and was shown not to correlate with serum LDH.^13^ Our observation was validated in a multicenter study conducted by Mansbach and colleagues, who also observed an inverse relationship between NW-LDH levels and bronchiolitis severity in young children presenting to the emergency department.^14^ In a bronchiolitis study conducted in the emergency department, Bennett and colleagues observed that an early robust proinflammatory immune response in the upper respiratory tract inversely correlated with duration of supplemental-oxygen therapy and did not contribute to severity of disease.^15^ In synthesizing the findings of these studies, we hypothesize that a major source of the lactate dehydrogenase in the nasal washes of children with bronchiolitis is likely derived from epithelial cells and/or neutrophils undergoing apoptosis as part of an innate immune response for controlling viral infection rather than from cellular necrosis of the epithelial cells and or inflammatory cells.

This prospective single-site cohort study of bronchiolitis extends our original observations, including a wider spectrum of disease severity: children presenting to the emergency center that are discharged home or admitted to either the general ward or an intensive care unit. We postulate that NW-LDH, NW-caspase 3/7 and the ratio of NW-LDH to NW-caspase 3/7 are predictive biomarkers of bronchiolitis severity, measured by disposition.

## Study design, materials and methods

### Study design

This was a prospective, cross-sectional single-site study evaluating healthy children less than 24 months of age presenting to the emergency department (ED) or the pediatric intensive care unit (ICU) with clinical bronchiolitis. Subjects were recruited from two separate and simultaneously occurring investigations (by both primary authors, RM and MS). Children were enrolled at Texas Children's Hospital from October 2010–April 2011, coinciding with the bronchiolitis season in Houston, TX. Infants with clinical bronchiolitis (wheezing and/or rales with a history of preceding upper airway illness), <24 months of age, and without significant co-morbidities were included. Patients excluded were greater than 24 months of age, had significant co-morbid medical conditions (for example, chronic lung disease, cyanotic congenital heart disease, neuromuscular disease, or immunodeficient state), were preterm (<36 weeks), or had respiratory distress unrelated to a viral URI.

Qualifying children were enrolled at the time they came to medical attention and sampled in the ED, or the ICU within 24 hours of admission, after obtaining informed consent from a parent or legal guardian. At enrollment, a physical exam was performed, demographic and medical history recorded, and nasopharyngeal secretion aspirate (NPA) obtained for viral diagnostics and biomarkers of interest. Enrollment, data collection, and sample collection for this study was done by the primary investigators (RM, MS), who were not involved in the medical care or disposition of subjects. Each child's disposition and outcome as determined by the primary care team was noted by review of the medical record. The Institutional Review Board of Baylor College of Medicine approved these studies.

### Sample collection

A single NPA sample was collected as previously described at enrollment, either in the emergency department or within 24 hours of admission to the ICU.^14^,^15^ NPA was used as a surrogate for the lower respiratory tract, as several studies confirm virus titers obtained in nasal washes correlate with disease activity in the lower airways.^16^ Briefly, NPAs were collected by instilling 2 cc of normal saline into the external nares, then aspirating the material into a 5-cc syringe containing an additional 2 cc of normal saline via a flexible catheter. Samples were stabilized with viral transport media (Iscove media with 15% glycerol) in a 1:1 dilution and refrigerated at 4 degrees before being transported to the Respiratory Virus Diagnostic Laboratory (CLIA ID 45D0919666) at Baylor College of Medicine. At the laboratory, specimens were divided into six aliquots of 0·5–1 cc and snap-frozen in an alcohol-dry ice bath and stored at −80°C. NPAs were generally processed within 24 hours of collection. Mucus presence in NPA specimens was evaluated weekly during enrollment by testing for the presence of secretory IgA (sIgA), a major immunoglobulin in mucosal defense, by a captured ELISA method using commercially available reagents 17,18. Only five NPA samples from the enrolled children demonstrated inadequate sIgA levels; these subjects were not resampled.

### Virus detection

Viruses in NPAs were identified by two methods: isolation from culture in four cell lines (rhesus monkey kidney, HEp-2, WI-38, and LLC-MK2), and real-time PCR (rt-PCR). For rt-PCR, specific primers and fluorescent probes were used to identify the following common viruses: RSV (A and B) rhinovirus (RV), parainfluenza viruses (1, 2, and 3), human metapneumovirus (HMPV), adenovirus (AD), influenza viruses (B, H3N2, and novel H1N1), enterovirus, and coronaviruses (229 E, OC43, NL63, and HKU1).^12^ Amplification of the gene for RNase-P, a ribonuclease enzyme, served as a positive internal control assessing the quality of samples. NPA samples with a cycle threshold value for RNase-P of less than 30 were considered optimal quality specimens; those between 30 to <35 were considered reasonable quality and those with ≥35 were of poor quality. Only two of the NPA samples were of poor quality for rt-PCR; the remaining were either optimal (*n* = 84) or reasonable (*n* = 45) quality specimens.

### Lactate dehydrogenase (LDH) quantification

LDH activity is a marker of cellular injury. LDH activity in the NPA samples was measured using a commercially available kit (Cytotoxic Detection Kit, Roche Applied Science, Indianapolis, IN, USA) per manufacturer instructions and as previously described.^13^ To calculate absolute values, a standard curve using L-LDH (Roche Applied Science) in serial dilutions was constructed demonstrating an ample linear dynamic range (*r = *0·996) at the dilutions tested (0·8–110 U/ml).

### Caspase 3/7 quantification

Caspase 3/7 activity is a marker of apoptosis. Caspase 3/7 activity was measured in NPA samples using the Caspase-Glo-3/7 kit (Promega, Madison, WI, USA). The assay generates luminescent activity proportional to caspase activity. Luminescence was measured with a FLUOstar OPTIMA microplate reader (BMG Labtech, Cary, NC, USA) and expressed in relative luminescence units. To calculate relative units (U/l) of caspase activity, a standard curve using recombinant human caspase-3 protein (R&D Systems, Minneapolis, MN, USA) was used demonstrating an ample linear dynamic range of activity at the dilutions tested (20 000–32 U/l). The value was expressed as relative units and it was assumed that one unit of caspase activity was equal to 0·1 ng of protein. It is important to note that caspase 3 activity can vary from lot to lot.

### Statistical analysis

The outcome used for primary analysis was disposition at the time of initial evaluation. The children were either discharged to home or after < 24-hour observation (home), admitted to an acute care unit (ACU), or admitted to a pediatric or neonatal intensive care unit (ICU). Demographic, clinical, virologic, and biomarkers were analyzed with respect to outcome. Ages of participants were categorized into four groups: 0–3 , 4–6 , 7–11 , and 12–23 months. Duration of illness prior to presentation was classified into three groups: 0–2, 3–5 and >5 days. Viral results were categorized as RSV, RV, RSV and RV co-infection, and all other viruses. Virus infection patterns were single viral infection, co- viral infection, and no virus identified. For descriptive statistics, continuous variables were represented as mean (or median), and standard deviation; categorical variables were represented as frequencies or percentages. Demographic characteristics among the groups were compared using chi- square test or Fisher's exact test. Analysis of the laboratory data for the continuous variables NW-LDH, NW-caspase 3/7, and NW-LDH/NW-caspase 3/7 ratio compared with demographic data (including disposition) was performed using analysis of variance (anova), Student's t- test, or Wilcoxon–Mann–Whitney tests as appropriate. Given the range of values, the log_10_-transformed values for NW-LDH and NW-caspase 3/7 activity were used for statistical analysis. Correlations were calculated using Pearson's or Spearman's coefficient. For the identification of independent factors that may influence disposition, multivariable logistic regression analyses (polytomous responses) were performed to calculate odds ratio and corresponding 95% confidence intervals. To identify independent factors that influenced NW-LDH, NW-caspase 3/7 and NW-LDH/NW-caspase 3/7 ratio, multiple linear regression models were developed with two-way interaction effects while controlling for age, gender, race, duration of illness, exposure to cigarette smoke, disposition of patients, virus infection pattern, and the relevant biomarkers. All statistical analyses were performed using the SAS software package version 9.2 (SAS Institute, Inc., Cary, NC, USA).

## Results

### Study population

In the 2010–11 respiratory viral season, we enrolled 131 children <2 years of age with bronchiolitis; 112 children were enrolled from the emergency department and 19 children were enrolled within 24 hours of admission to the pediatric ICU. The average age (mean ± SD) of children was 7·2 ± 5·8 months. Most children (51·9%) were Hispanic, followed by African-American (24·2%) and white (22·9%). Of enrolled children, 58·8% were male. Most children (64·9%) had been breastfed, and just over half (51·9%) presented on days 3–5 of illness. Only 26·9% of children attended daycare, and 33·6% of children lived in homes with smokers. A diagnosis of asthma was found in 8·4% of children. Demographic data of the participants stratified by disposition (home, ACU, ICU) are presented in Table 1. There was no difference among groups in terms of age, gender, race, or asthma diagnoses; the groups were also similar in terms of exposure to daycare, tobacco smoke, and breastfeeding. The only demographic variable that differed significantly was duration of illness prior to presentation: children presenting on days 3–5 of illness were significantly likelier to be admitted to the ACU or ICU (*P* = 0·03).

**Table 1:** Demographic data of participants

Risk Factors	Disposition				*P*-values*
	All *n* = 131 (%)	Home + OBS *n* = 64 (%)	ACU *n* = 40 (%)	ICU *n* = 27 (%)	
Age (categories)					
0–3 months	40 (30·5)	14 (21·8)	19 (47·5)	7 (25·9)	0·18
4–6 months	31 (23·7)	17 (26·6)	6 (15·0)	8 (29·6)	
7–12 months	30 (22·9)	16 (25·0)	7 (17·5)	7 (25·9)	
>12 months	30 (22·9)	17 (26·6)	8 (20·0)	5 (18·6)	
Gender					
Males	77 (58·8)	38 (59·4)	23 (57·5)	16 (59·3)	0·98
Race					
Hispanic	68 (51·9)	34 (53·1)	22 (55·0)	12 (44·4)	0·94
White	30 (22·9)	14 (21·9)	8 (20·0)	8 (29·6)	
African-American	32 (24·4)	15 (23·4)	10 (25·0)	7 (25·9)	
Other	1 (0·8)	1 (1·6)	0 (0·0)	0 (0·0)	
Duration of Illness					
0–2 days	23 (17·6)	14 (21·9)	6 (15·0)	3 (11·1)	0·03
3–5 days	68 (51·9)	24 (37·5)	27 (67·5)	17 (63·0)	
>5 days	40 (30·5)	26 (40·6)	7 (17·5)	7 (25·9)	
Breast Fed					
Yes	85 (64·9)	43 (67·2)	28 (70·0)	14 (51·9)	0·27
Diagnosis of Asthma					
Yes	11 (8·4)	6 (9·4)	2 (5·0)	3 (11·1)	0·63
Day care					
Yes	35 (26·9)	19 (29·7)	8 (20·5)	8 (29·6)	0·56
Exposure to cigarette smoke					
Yes	44 (33·6)	22 (34·4)	10 (25·0)	12 (44·4)	0·25

* Chi-square test or Fisher's exact test.

### Virology

Nasal wash served as a surrogate marker for lower respiratory tract infection.^16^ Viral pathogens were detected in 91·6% of children, consistent with previous studies.^13^,^15^ Of 131 patients, a single infection was found in 81 (61·8%); 39 (29·8%) were co-infected with more than one virus. The most commonly detected viruses were RSV(*n* = 84; 64·1%) and RV (*n* = 34; 26·0%). Other common respiratory viruses identified included human metapneumovirus (HMPV) (*n* = 8; 6·1%), influenza (*n* = 5; 3·1%), parainfluenza (*n* = 1; 0·8%), cytomegolovirus (CMV) (*n* = 7; 5·3%), adenovirus (*n* = 9; 6·9%), coronavirus (*n* = 6; 4·6%) and enterovirus (*n* = 1; 0·8%). As shown in Table 2, neither virus infection type nor virus infection pattern impacted disposition. There was also no difference in disposition between infants infected with RSV/A versus RSV/B (data not shown).

**Table 2:** Viral pathogen and infection pattern by disposition

Virus variables	Disposition				*P*-value
	All	Home + OBS	ACU	ICU	
Virus infection type					
RSV only	66 (50·4)	34 (53·1)	19 (47·5)	13 (48·2)	0·53
HRV only	16 (12·2)	9 (14·1)	3 (7·5)	4 (14·8)	
RSV + HRV	18 (13·7)	5 (7·8)	8 (20·0)	5 (18·5)	
Others	31 (23·7)	16 (25·0)	10 (25·0)	5 (18·5)	
Virus infection pattern					
Single	81 (61·8)	41 (64·1)	22 (55·0)	18 (66·7)	0·77
Co-infection	39 (29·8)	19 (29·7)	13 (32·5)	7 (25·9)	
No virus detected	11 (8·4)	4 (6·2)	5 (12·5)	2 (7·4)	

### Biomarkers

**Figure 1:**
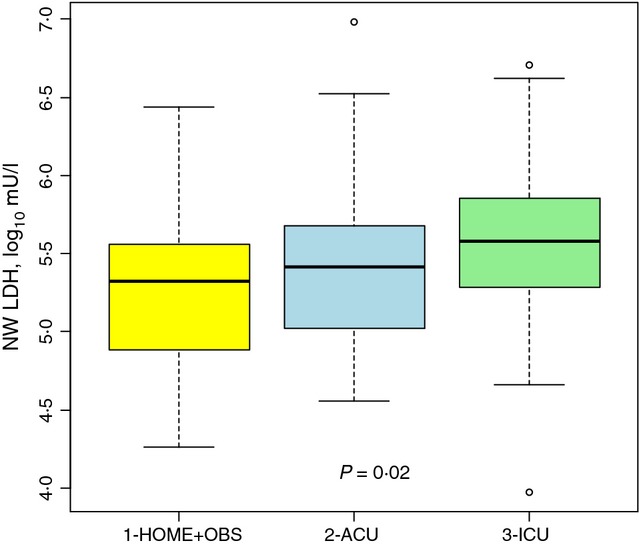
LDH in Nash Wash (log_10_ mU/l) according to the three categories of disposition: HOME + OBS, ACU, and ICU.

**Figure 2:**
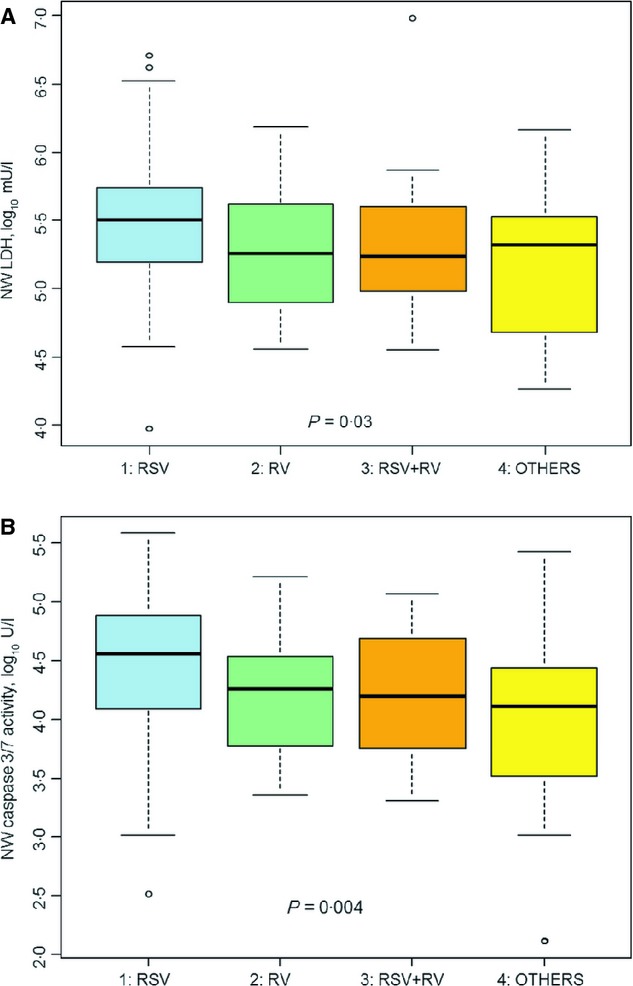
(A) LDH in Nasal Wash ((log_10_ mU/l) Compared to Different Viruses. (B) Caspase 3/7 Activity in Nasal Wash compared to Different Viruses.

NW-LDH, NW-caspase 3/7, and NW-LDH/NW-caspase 3/7 ratio were normalized by log_10_ transformation and analyzed in terms of disposition. NW-LDH varied significantly among the three disposition groups, as shown in Figure 1. Children admitted to an ICU had significantly higher NW-LDH (log_10_ mU/l; mean ± SD, 5·56 ± 0·58) than children who were sent home (mean ± SD 5·23 ± 0·49) or admitted to the ACU (mean ± SD 5·39 ± 0·50). NW-LDH also varied significantly by virus type, as illustrated in Figure 2. Children infected with RSV alone had the highest NW-LDH concentration (log_10_ mU/l; mean ± SD, 5·47 ± 0·49) compared with those co-infected with both RSV and RV (5·34 ± 0·54), with RV only (5·27 ± 0·46) or another virus type (5·14 ± 0·56). However, univariate analysis indicated that singly infected patients did not have significantly higher NW-LDH when compared with co-infected patients or those with no virus identified, although there was a trend toward higher NW-LDH in singly infected patients (data not shown).

**Figure 3:**
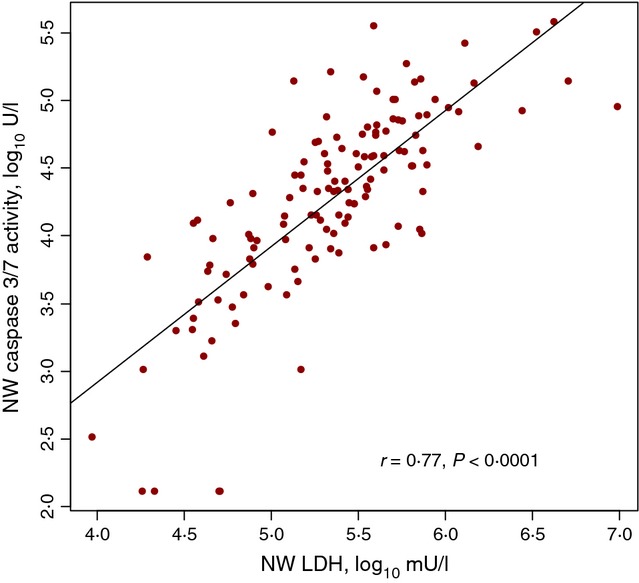
Correlation of LDH to caspase 3/7 Activity in Nasal Wash.

As has been demonstrated previously,^13^ there was a strong correlation (*r* = 0·77, *P *≤ 0·0001) between NW-LDH and NW-caspase 3/7 activity, illustrated in Figure 3. Interestingly, NW-caspase 3/7 activity differences among the disposition groups were not significant (*P* = 0·4). However, NW-caspase 3/7 activity varied significantly by virus type (RSV only, RV only, RSV + RV or others, *P* = 0·004), as seen in Figure 2B. Those patients infected with RSV only had the highest caspase level in their nasal washes (mean ± SD, 4·48 ± 0·58), compared with those co-infected with both RSV and RV (4·21 ± 0·54), RV only (4·17 ± 0·52) or other types of viruses (3·91 ± 0·88). In addition, the NW-LDH/NW-caspase 3/7 ratio difference between the disposition groups did not achieve statistical significance (*P* = 0·07). However, when the children were divided into two disposition groups, hospitalized or not, the NW-LDH to NW-caspase 3/7 ratio in the hospitalized group was significantly higher (mean ± SD, 1·15 ± 0·48, median, 1·15) than those children not hospitalized (1·01 ± 0·39, median 0·98, *P* = 0·03).

Multivariable logistic regression analyses were conducted using disposition of the patients (home, ACU, and ICU) as the primary outcome to identify demographic and biomarker variables that contributed to disposition. The analysis demonstrated that (i) Children younger than 3 months were more likely to be admitted to the ACU (OR: 3·87, 95% CI: 1·11–13·50) versus being sent home compared to children older than 12 months; (ii) Compared with children who presented on day 6 of illness or later, children presenting on days 3–5 of symptoms were significantly more likely to be admitted to either the ACU (OR: 4·07, 95% CI: 1·34–12·36) or ICU (OR 3·69, 95% CI: 1·14–11·90); (iii) A one-unit increase in NW-LDH (log10, mU/l) was associated with an increased likelihood of admission to either the ACU (OR: 6·68, 95% CI: 1·53–29·29) or ICU (OR 6·10, 95% CI: 1·28–28·99); (iv) By contrast, a one-unit increase in NW-caspase 3/7 activity (log10, U/l) was associated with a decreased likelihood to be admitted to the ACU (OR: 0·33, 95% CI: 0·11–0·96).

Three multiple linear regression models were constructed to evaluate the demographic, clinical and virologic variables impacting our biomarkers of interest (Table 3). The independent variables and interaction terms providing the best R-square value were used to construct the models. R-squared values indicate that the models explained 73·2%, 72·2%, and 32·0% of the variation for NW-LDH, NW-caspase, and NW-LDH/caspase ratio, respectively. The β coefficient and standard error are shown for each of the independent variables; an asterisk indicates significant variables. The results indicate male children had higher NW-LDH compared with females; Hispanic children had lower NW-LDH and NW-LDH/caspase 3/7 ratio compared to non-Hispanic children; higher NW-LDH and NW-LDH/NW-caspase 3/7 ratio was significantly associated with ACU admission; NW-caspase 3/7 activity was inversely associated with ACU admission; NW-LDH was significantly lower, and NW-caspase 3/7 significantly higher, in children with an identifiable pathogen; and NW-LDH was highly associated with NW-caspase 3/7.

**Table 3:** Multiple Linear Regression Models for NW-LDH (log10 mU/l), NW-Caspase 3/7 (log10 U/l), and NW-LDH/NW-Caspase 3/7 ratio

Predictors	β (SE) for NW-LDH	β (SE) for NW-caspase 3/7	β(SE) for NW-LDH/NW-caspase 3/7 ratio
Age (categories)			
≤3 months	0·062 (0·114)	0·007 (0·152)	−0·019 (0·152)
4–6 months	0·050 (0·109)	−0·019 (0·145)	0·011 (0·145)
7–12 months	0·225 (0·106)*	−0·256 (0·143)	0·242 (0·142)
>12 months	Reference	Reference	Reference
Gender			
Males	0·117 (0·059)*	−0·134 (0·079)	0·127 (0·078)
Females	Reference	Reference	Reference
Hispanic			
Yes	−0·674 (0·201)**	0·976 (0·266)**	−0·959 (0·265)**
No	Reference	Reference	Reference
Disposition			
ACU	0·371 (0·139)**	−0·424 (0·188)*	0·402 (0·186)*
ICU	0·129 (0·167)	0·040 (0·224)	−0·069 (0·221)
Home + OBS	Reference	Reference	Reference
Duration of Illness			
<3 days	−0·132 (0·087)	0·079 (0·117)	−0·063 (0·116)
3–5 days	−0·036 (0·067)	0·025 (0·090)	−0·021 (0·090)
>5 days	Reference	Reference	Reference
Exposure to cigarette smoke			
Yes	−0·196 (0·085)*	0·166 (0·115)	−0·148 (0·113)
No	Reference	Reference	Reference
Virus infection pattern			
Single	−0·445 (0·175)*	0·917 (0·224)**	−0·936 (0·223)**
Co-infection	−0·458 (0·181)*	0·865 (0·234)**	−0·877 (0·234)**
No virus detected	Reference	Reference	Reference
Log10 (NW-CASPASE 3/7)	0·600 (0·043)**	–	–
Log10 (NW-LDH)	–	1·068 (0·077)**	–
Agecat*Disposition			
<3 m and ACU	−0·296 (0·170)	0·246 (0·229)	−0·218 (0·226)
<3 m and ICU	0·137 (0·217)	−0·096 (0·290)	0·081 (0·289)
4–6 m and ACU	−0·531 (0·203)*	0·595 (0·273)*	−0·562 (0·270)*
4–6 m and ICU	−0·067 (0·210)	−0·122 (0·280)	0·149 (0·278)
7–12 m and ACU	−0·671 (0·193)**	0·933 (0·257)**	−0·911 (0·256)**
7–12 m and ICU	−0·042 (0·213)	0·199 (0·283)	−0·214 (0·282)
Exposure to cigarette smoke*Disposition			
Yes and ACU	0·442 (0·144)**	−0·455 (0·196)*	0·423 (0·193)*
Yes and ICU	0·140 (0·164)	−0·365 (0·217)	0·379 (0·216)
Hispanic*Virus infection pattern			
Yes and single	0·735 (0·216)**	−1·143 (0·282)**	1·133 (0·282)**
Yes and co-infection	0·697 (0·228)**	−0·971 (0·303)**	0·949 (0·302)**
Model's R-square	0·732	0·722	0·320

Log10-transformed values were used in the analyses for NW-LDH (log10 mU/l), NW-Caspase 3/7 (log10 U/l), and NW-LDH/NW-Caspase 3/7 ratio.

* *P* < 0·05;

** *P* < 0·01.

When accounting for interaction terms, several independent variables had modifying effects on other independent factors, which may be of important consideration when determining the severity of bronchiolitis. For example, children 4–12 months old hospitalized in the ACU actually had lower NW-LDH and NW-LDH/NW-caspase 3/7 ratio than children aged ≥12 months that were sent home. Also, children exposed to second-hand cigarette smoke hospitalized in the ACU had higher NW-LDH and NW-LDH/NW-caspase 3/7 ratio than those sent home living with non-smokers. Hispanic patients infected with single virus or co-infected with multiple viruses had significantly higher NW-LDH, lower NW-caspase, and higher NW-LDH/NW-caspase 3/7 ratio than those with no virus identified and compared with other races (*P* < 0·01).

## Discussion

In this study, we identify added complexity in using the promising biomarker NW-LDH for children with bronchiolitis. As an intracellular enzyme released upon cell injury, NW-LDH can be a marker of necrosis or apoptosis. Similar to our prior study,^13^ RSV infection was associated with higher NW-LDH, and NW-LDH significantly correlated with NW-caspase 3/7. Unlike our prior studies where children with values in the upper quartile had a decreased risk of hospitalization,^13^,^14^ in this study an increasing concentration of NW-LDH was associated with an increasing likelihood of hospitalization to either the ACU or ICU. Children admitted to an ICU had significantly higher NW-LDH than children who were sent home or admitted to the ACU. These differences in predicting outcome are not readily explained; however, a higher proportion of this cohort required ICU care, (20·6% versus 6·1%) and none of the children had major co-morbidities compared with 25·2% in Laham *et al*. study.^13^ It is possible that our current cohort of children had more severe bronchiolitis, resulting in greater cellular injury to the airway epithelium. Thus, understanding the mechanisms (apoptosis versus necrosis) underlying the release of LDH might improve the prediction of outcomes, with the implication that NW-LDH from cells undergoing dysregulated inflammatory and necrotic processes results in more severe disease while NW-LDH originating from controlled apoptotic antiviral processes results in less severe disease.

To further address this issue, we evaluated caspases 3 and 7, as caspases play a vital role in, and are biomarkers for, apoptosis. Caspases 3 and 7 are effector caspases that break down intracellular proteins and trigger the apoptotic pathway. We reasoned that children with greater NW-caspase 3/7 activity were more likely to have controlled antiviral pro-inflammatory responses with less severe illness. Previous studies have shown a role for both granulocyte and respiratory epithelial cell apoptosis in antiviral immunity.^19^ Further, some investigation has shown that incubating RSV with granulocytes inhibits apoptosis *in vitro*, while a study of RSV positive infants has shown increased granulocyte apoptosis *in vivo*.^20^,^21^ Other studies have highlighted that epithelial cell apoptosis is rare in early disease,^22^ but predominates in later phases of illness.^23^ These results suggest appropriate regulation of the apoptotic response is a complex process and influences the severity of disease. In the multivariable logistic regression analyses, we observed that a one-unit increase in NW-caspase 3/7 activity (log10, U/l) was associated with a decreased likelihood to be admitted to the ACU (OR: 0·33, 95% CI: 0·11–0·96). This was supported with the NW-LDH/NW-caspase 3/7 ratio: children going home from the ER had significantly lower NW-LDH/NW-caspase 3/7 ratios compared with hospitalized children (mean ± SD: 1·01 ± 0·39 versus 1·15 ± 0·48 *P* = 0·03), possibly reflecting a more balanced innate immune response to the viral pathogen. The relationship between NW-LDH and NW-caspase 3/7 is a crucial finding and suggests that NW-LDH or NW-caspase 3/7 alone may not be sufficient markers, but together can help predict severity of bronchiolitis.

The multiple linear regression analyses demonstrated that some interaction effects between demographic variables may be of such significance they can change or predict the severity of bronchiolitis. Our univariate models showed that higher NW-LDH and NW-LDH/NW-caspase 3/7 ratio were strongly linked to more severe illness and hospitalization to the ICU and ACU. Interestingly, when multiple linear models were used for the analyses, the age of the subjects in different levels of disposition also greatly impacted the changes of biomarkers. Those children 4–12 months of age who were hospitalized to the ACU actually had a lower NW-LDH than those sent home whose ages were greater than 12 months, which had the same trend as the results in our previous studies.^13^,^14^ Thus, the demographic interactions should be considered in conjunction with biomarker data when determining severity of illness. Although this study was not aimed toward explaining these underlying associations, a single variable alone may not be sufficient enough to explain what drives the changes of NW-LDH, NW-caspase 3/7, and NW-LDH/NW-caspase 3/7 ratio.

As previously mentioned, our earlier studies demonstrated an inverse relationship between NW-LDH and bronchiolitis disease severity with children in the top quartile having a significantly lower risk of being hospitalized for bronchiolitis.^13^,^14^ In this study, the univariate analysis appears to contradict those previous reports. However, this report included a group of children who were older, healthier at baseline, presenting with more varied degree of illness, and had a greater proportion requiring a high level of care. Also, our samples were processed after only one freeze–thaw cycle; samples used for the earlier study underwent multiple freeze–thaw cycles, which may have adversely affected stability of the LDH isoenzymes and or quality of the samples. In our current study, we ensured the quality of our samples by providing feedback in real time on the detection of sIgA in the nasal wash fluid and using nasal wash samples thawed only once for determining LDH and caspase 3/7 activity. Also, in our previous reports, data on NW-LDH/NW-caspase 3/7 ratio was not performed, which we believe helps to explain the dominant cellular pathway (necrosis versus apoptosis) from which LDH derives in respiratory tract fluid. As previously stated, our multiple linear models showed the same inverse relationship between NW-LDH (or NW-LDH/NW-caspase 3/7 ratio) and the severity of bronchiolitis for the children who were admitted to ACU compared with those sent home when taking into account the age of the patients. Overall, our observations over time support the concept that biomarkers such as NW-LDH and NW-caspase 3/7 reflect a complex pathway for control of respiratory viral infections and bronchiolitis disease severity.

Further work exploring the balance between necrosis and apoptosis has been done in in-vitro studies showing that virally infected human lung epithelial cells release both LDH and caspases.^24^ Additional studies would likely include further clinical work with hospitalized infants over time, demonstrating the overall trajectory of these biomarkers relative to clinical status.

A limitation of our study is that it was performed at one site during one respiratory virus season and thus the findings may not be applicable to other regions with differences in demographics and viral epidemiology. In addition, subjects were recruited as part of two originally separate studies; those subjects recruited from the ICU were sampled within the first 24 hours of admission, but all other patients were enrolled and sampled at the time of presentation to the ER. This may have impacted on NW-LDH activity with higher concentrations detected later into the acute phase of the illness. This, in part, is supported by a significantly higher proportion of children admitted to the ACU on days 3–5 of illness compared with children who were discharged home from the emergency department.

In conclusion, NW-LDH, NW-caspase 3/7 and the NW-LDH/NW-caspase 3/7 ratio when used in combination can help predict outcome in children with bronchiolitis. Understanding the underlying relationship between LDH, caspase, and the innate immune response to respiratory viral pathogens will likely strengthen this predictive ability. Demographic variables and their interaction effects must also be considered when explaining the biomarkers of interest with respect to outcomes and disposition. The ratio of NW-LDH/NW-caspase 3/7 reflects the innate immune balance between necrosis and apoptosis in controlling disease severity in children presenting with bronchiolitis. Our observations support the hypothesis that during the early immune response to control acute viral respiratory tract infection, an excess of necrosis of epithelial cells from either direct viral replication or a secondary inflammatory response is detrimental to the host versus a regulated apoptotic, antiviral pro-inflammatory pathway of epithelial cells designed to inhibit viral replication and progression of airway injury. Biomarkers like NW-LDH and NW-caspase 3/7 that can help elucidate the pathogenesis of virally induced airway disease will likely serve as good predictive biomarkers in the clinical setting. Further study of LDH, caspase and other biomarkers may reveal novel targets for diagnostic tools that would aid clinicians in management and disposition of children with bronchiolitis.
